# Use of a knowledge broker to establish healthy public policies in a city district: a developmental evaluation

**DOI:** 10.1186/s12889-016-2832-4

**Published:** 2016-03-15

**Authors:** Kirsten Langeveld, Karien Stronks, Janneke Harting

**Affiliations:** Department of Public Health, Academic Medical Center, University of Amsterdam, P.O. Box 22700, Amsterdam, 1100 DE The Netherlands

**Keywords:** Knowledge brokering, Agenda-setting, Alternative specification, Healthy public policies, Boundary spanning, Ripple effect, Participant observation method

## Abstract

**Background:**

Public health is to a large extent determined by non-health-sector policies. One approach to address this apparent paradox is to establish healthy public policies. This requires policy makers in non-health sectors to become more aware of the health impacts of their policies, and more willing to adopt evidence-informed policy measures to improve health. We employed a knowledge broker to set the agenda for health and to specify health-promoting policy alternatives. This study aimed at gaining in-depth understanding of how this knowledge broker approach works.

**Methods:**

In the context of a long-term partnership between the two universities in Amsterdam and the municipal public health service, we employed a knowledge broker who worked part-time at a university and part-time for an Amsterdam city district. When setting an agenda and specifying evidence-informed policy alternatives, we considered three individual policy portfolios as well as the policy organization of the city district. We evaluated and developed the knowledge broker approach through action research using participant observation.

**Results:**

Our knowledge brokering strategy led to the adoption of several policy alternatives in individual policy portfolios, and was especially successful in agenda-setting for health. More specifically, health became an issue on the formal policy agenda as evidenced by its uptake in the city district’s mid-term review and the appointment of a policy analyst for health. Our study corroborated the importance of process factors such as building trust, clearly distinguishing the knowledge broker role, and adequate management support. We also saw the benefits of multilevel agenda-setting and specifying policy alternatives at appropriate policy levels. Sector-specific responsibilities hampered the adoption of cross-sectoral policy alternatives, while thematically designed policy documents offered opportunities for including them. Further interpretation revealed three additional themes in knowledge brokering: boundary spanning, a ripple effect, and participant observation.

**Conclusions:**

The employment of a knowledge broker who works simultaneously on both agenda-setting for health as well as the specification of health-promoting policy alternatives seems to be a promising first step in establishing local healthy public policies. Future studies are needed to explore the usefulness of our approach in further policy development and policy implementation.

## Background

Public health is to a large extent determined by non-health-sector policies, as many environmental, economic and political factors influencing health are beyond the authority of the health sector itself [1]. One approach for addressing this seeming contradiction is to build healthy public policies [2]. Building healthy public policy has been referred to as putting “…health on the agenda of policy makers in all sectors and at all levels, directing them to be aware of the health consequences of their decisions and to accept their responsibilities for health” [3]. Despite the ample attention the approach has received over the last decades [4], establishing healthy public policies remains challenging [1]. One barrier to the establishment of such policies is insufficient awareness among the actors involved of the potential health impacts of non-health-sector policies—whether negative or positive [5, 6]. Another barrier is that evidence in support of health-promotion measures is not easily accepted and integrated into the policies of other sectors [7]. Although a knowledge broker could be beneficial in this respect [8–10], evidence on how knowledge is effectively disseminated and translated across boundaries between research and policy remains anecdotal and inconclusive [9, 11, 12].

We define a knowledge broker as an intermediary between the world of research and that of policy and practice [13]. Knowledge is usually brokered between producers (e.g. public health scientists) and users (e.g. public health policy makers) [11, 14]. In this respect, a knowledge broker makes strategic use of information and has the competency to influence others by presenting them with models and ideas based on scientific knowledge [15]. Conceptually, three frameworks for—or dimensions of—knowledge brokering can be distinguished [12, 15]: The first framework is knowledge management, which concerns the creation, diffusion and uptake of knowledge. The second framework is linkage and exchange [13], which includes connecting researchers and decision makers and facilitating interaction amongst them. Thirdly, capacity building, either individual or organizational, refers to developing the ability to interpret and use research evidence [15]. As these practices often occur together [12], knowledge brokering is likely to include a broad range of activities [9, 11, 16], such as identifying problems; selecting, interpreting and communicating knowledge; motivating producers and users; mediating between stakeholders; and providing instruction to individuals for the integration of knowledge in their policies. The knowledge broker role, whether performed by individuals, groups or organizations, can therefore be considered challenging and requiring different kinds of expertise and a broad range of skills [16]. Hence, knowledge brokering is a complex social activity [12] that is likely to vary according to its context [11]. This complexity makes knowledge brokering difficult to evaluate [17]: it is not clear what types of outcomes should be assessed (i.e. interactions between producers and users, improved capacity for using evidence, or actual increased evidence use); nor is it clear how these outcomes can be adequately captured (e.g. via surveys, interviews or documentary analyses).

To enhance the creation of evidence-based healthy public policies in a city district in Amsterdam, the Netherlands, a knowledge broker was employed for a period of 3 years (2011–2013). In order to address the lack of awareness and disregard for evidence concerning this policy approach, the knowledge broker worked on both agenda-setting for health and the specification of health-promoting policy alternatives [18, 19]. Given the context-specificity of knowledge brokering [11] and the uncertainties regarding appropriate assessments of the approach, we decided to evaluate the 3-year pilot using participant observation [20, 21]. This method was also the most suitable for gaining the in-depth understanding of the knowledge broker approach that is currently required [12]. The aim of our study was therefore to gain in-depth understanding of how the knowledge broker approach works, what its effects are, and to identify the conditions under which such effects appear.

## Methods

### Context

The pilot was developed by the academic collaborative center (ACC) for public health in Amsterdam. This ACC is a long-term partnership between the municipal public health service (PHS) and several university departments at the city’s two academic medical centers. The main purpose of an ACC is to improve the exchange of knowledge between policy makers, researchers and practitioners [22]. As achieving this purpose in relation to healthy public policies was likely to be challenging, it was decided to start the knowledge broker pilot in a single city district of Amsterdam. At that time, city districts served as independent administrative and policy units. The deprived city district we selected for the pilot had 139,000 inhabitants [23]. Common health-related problems in this district included chronic conditions, such as cardiovascular diseases and psychosocial problems (e.g. depression and anxiety), which were accompanied by risk factors related to behavior and the environment (e.g. smoking, overweight, stress, nuisance and pollution) [24]. The city district’s recent participation in a “Healthy Neighborhood Experiment” had made health a policy issue in a corner of this city district and created an initial willingness to engage in a subsequent pilot on healthy public policies. Agreement to participate in the knowledge broker pilot was reached in a preparatory meeting attended by an alderman and a senior civil servant from the city district, a department head at the PHS, and a senior public health scientist at the university.

### Knowledge broker

#### Background

As it is recommended that knowledge brokers “possess expertise from both end users’ and researchers’ domains” [16], we appointed a postdoctoral anthropologist with 3 years’ work experience as a civil servant in another Amsterdam city district [first author KL]. In this way the knowledge broker acted as a boundary spanner. A boundary spanner “links tw**o** or more systems whose goals and expectations are likely to be at least partially conflicting” [42: 77).

#### Positioning

The knowledge broker worked half-time at the city district’s department of Welfare, Education and Employment and half-time at one of the universities’ departments of public health. Like all city district employees, she was given an access account (providing her with an internal email address, a key to all doors and access to the intranet), and she signed the Formal Secrecy Act, binding her to secrecy. She attended regular meetings, took part in corridor chats, and shared a room with a senior civil servant who introduced her to his network. This positioning gave her access to significant stakeholders and policy-related and contextual information. To support her knowledge brokering activities (as mentioned above in the Introduction), she could consult public health scientists at both universities as well as public health specialists from the PHS. The fact that she was also employed by the university gave her the opportunity to critically reflect on the knowledge broker role from a scientific perspective. In order to monitor the quality of the pilot and its evaluation, representatives from all organizations involved were on either the pilot’s project team, or the pilots’ advisory board, or both.

#### Approach

Our approach included both agenda-setting for health and the specification of health-promoting policy alternatives. Agenda-setting [18] included creating awareness of health as an important policy theme, viewed separately as well as in relation to other policies. For that purpose, the “rainbow model” was used [25]. This model shows that health is not just determined by individual factors, but also by environmental and socioeconomic determinants, and that these are especially influenced by policies in other domains.

To specify alternative policy options [22], the knowledge broker first made an analysis of relevant policy documents. She checked whether health was a theme and—mostly together with a senior health scientist [last author JH]—to what extent noticeable determinants of health were optimally addressed. Second, by reviewing the literature herself and/or by consulting scientific experts, the knowledge broker searched for evidence-informed policy strategies that could improve the city district’s position on the determinants in question, in order to optimize public health in the long-term. Third, specific policy alternatives were formulated.

#### Procedure

In the city district, the knowledge broker followed two parallel pathways: one that included three individual policy portfolios and one that encompassed the entire policy organization of the city district.

The individual policy portfolios were selected after reviewing the city district’s administrative agenda for the policies that were to be developed during the current term (2010–2014). The policies regarded most suitable for the pilot were the policy on Lesbian, Gay, Bisexual and Transgender persons (LGBT), and the policies on Poverty and on Economy. There were three main reasons for selecting these policies. Firstly, all three were mainstream policies still in the making, and addressed relevant environmental or socioeconomic determinants of health. Secondly, their policy makers offered an initial opportunity for agenda-setting. Thirdly, the knowledge broker felt sufficiently equipped to specify alternatives. The responsible civil servants were invited by email, by telephone or in person to attend a first individual meeting in the city district during which the pilot was explained and the agenda for health was set. Policy alternatives for integrating health and specific measures on determinants of health were discussed in one or more follow-up meetings in the city district or at another convenient location. Most of these individual or small-group meetings took about an hour.

With regard to the entire policy organization of the city district, several small-group or medium-group meetings were organized. These meetings had four main aims: agenda-setting for health in general, organizing management support for the knowledge broker’s activities, facilitating progress in the individual policy portfolios, and discussing policy alternatives to secure the long-term position of health on the city district’s policy and political agenda. Depending on their purpose, the meetings were attended by relevant representatives of policy, science and practice. City district participants included civil servants, senior civil servants, middle managers, a senior manager, and two aldermen. Among the PHS delegates were a head of department and some senior public health policy experts. University participants included two public health professors [including coauthor KS] and three senior public health scientists. Most of the group meetings took place in the city district and lasted between one and 4 h.

### Study

#### Design

We evaluated the knowledge broker pilot in a case study, using action research to explore the performance of the knowledge broker in depth. Action research has been defined as a flexible spiral process that allows action (i.e. change) and research (i.e. understanding) to be achieved at the same time [26]. The fact that the people affected by a change are usually themselves involved in action research makes it more likely that any understanding that results will be widely shared and the change pursued with commitment [26:2]. We opted for a developmental perspective in our evaluation method. This evaluation method allowed us to change the knowledge broker’s positioning, approach and procedure during the course of the pilot [27, 28]. This meant that we as researchers were required to work closely with the employees of the city district and the PHS. It is important that action research and developmental evaluation be seen as compatible methods [29]. That is, both inquiry methods follow a similar spiral learning process that alternates between action and critical reflection. Also, in order to gradually refine the methods, throughout the process attention must be given to the data and its interpretation [26]. However, whereas the focus of action research is on solving problems through creating change, the primary aim of developmental evaluation may be more on judging and adapting the innovative change process as such [29]. Both inquiry methods are therefore also considered to be both complementary and mutually reinforcing [29]. Combining the two methods enabled us “to adapt existing policies to new conditions in a complex dynamic system” [29:21] and—whenever needed and wherever possible—to feed back our findings for immediate use in the pilot. For these purposes, the role of knowledge broker and action researcher was combined in the hands of one person.

#### Data collection

Data was collected using the ethnographic method of participant observation [20]. This implies that the researcher, as knowledge broker, was an active participant in the city district’s policy process [30], while at the same time she systematically observed the developments within this context [31]. As is common in this type of research, key data for the study was comprised of field notes of observations taken by the knowledge broker. These field notes concerned all formal and informal meetings the knowledge broker had been engaged in. Such notes reported for instance who was involved, what content was discussed, how agenda-setting activities were received, the extent to which policy alternatives were adopted (if at all), and what the contextual developments were.

#### Data analysis

Data was analyzed in two main ways. Firstly, the field notes were written up as “thick descriptions” [32]. Since field notes not only contain data and analyses but are also a record of occurrences in the field, they are always a construction of the researcher [20]. The field notes were additionally organized into category folders, for a thematic ordering of the data. Secondly, a journal was compiled, comprising a chronological account of occurrences, including the reflections of the researcher. Additional data sources were materials the knowledge broker had used to draw up her approach (see above), such as local policy documents, websites on health statistics and evidence-informed policy measures, scientific literature and expert reports, as well as materials that had been part of her procedure (see above), such as email correspondence, presentations and minutes of meetings. The first and third author qualitatively analyzed these data. In participant observation, fieldwork and analysis are not separate activities − they form an iterative process in which interpretation moves from findings to ideas and theory, then back to the field again [31]. This iterative process was used to analyze the data. Trustworthiness was warranted by discussing the same issues with different informants from the policy and management domains, as well as by discussing our observations and interpretations with the key informant [20, 33]. For each of the individual policy portfolios and the district’s policy organization as a whole, this analysis resulted in a report containing the following four components [12]: (I) key characteristics of current policies, (II) key activities of the knowledge broker, (III) key impacts in terms of agenda setting and policy development, and (IV) reflections on the key process factors in this respect.

### Ethical approval

According to provisions of the Dutch Medical Research Involving Human Subjects Act, this study did not require approval to carry out the research from a medical research ethics committee in the Netherlands [34]. Oral permission to carry out the research was received from the administration, management and policy analyst. This oral approval was given before participating in the research. Participants were informed about the research and its goals at the start of the first meeting. Confidentiality and anonymity was ensured. Because our results section contains detailed descriptions of various occurrences, we asked the key informant of the city district to confirm that confidentiality and anonymity had been sufficiently taken care of, also in order to prevent our manuscript harming any of the participants. The key informant thought that the ethical question of anonymity of the participants had been well taken care of.

## Results

### Portfolio 1: Policy on LGBT persons

July − September 2011

I Current policy

The policy document on Lesbian Gay Bisexual Transgender persons (LGBT) concentrated on the safety and liberty of this minority population in the city district. It also reported on specific mental health problems—such as “minority stress”—resulting from discrimination and restrictions in expressing ones identity, and outlined specific organizations that LGBTs could consult in this respect.

II Activities of the knowledge broker

After a first departmental meeting to introduce the pilot, one civil servant asked for advice on how to write policy on LGBT health. In a one-to-one meeting, the knowledge broker further explained how environmental and socioeconomic determinants affect LGBT health. She also commented on non-health-related issues in the LGBT policy document as well as on the budget available. The civil servant subsequently and unexpectedly terminated the collaboration by email. Since no policy alternatives could therefore specified, the knowledge broker organized a timely follow-up meeting to find out the civil servant’s reason for ending the collaboration.

III Impact on agenda and policies

After the one-to-one meeting, the civil servant initially expressed an interest in receiving more information on the health of LGBTs. This person’s reason for subsequent termination of the contact was that they were not convinced of the added value of the knowledge broker’s advice.

IV Reflections on process factors

#### Building rapport

The civil servant did share critical reflections on the city district’s procedures with the knowledge broker, demonstrating that initial rapport had been built. Building rapport, or “relationship building” has been reported to be an important part of successful knowledge brokering [9]. Nevertheless, it was also felt that the contact may also have been terminated due to insufficient confidence in the knowledge broker as a reliable source of policy alternatives. Hence, it was decided that building rapport deserved more attention in subsequent portfolios. Building a ‘strong relationship with “users”’ is mentioned as a prerequisite for being ‘effective’ as a knowledge broker [15].

#### Role distinction

By commenting on the content and budget of LGBT policy, the knowledge broker assumed the role of civil servant, which happened rather accidentally because of her own background as a civil servant. Such a crossing of role boundaries may also have triggered the decision to terminate the contact. Different sources of such insider-outsider tensions have been described previously by Minkler [35]. Suddenly having another “civil servant” on the LGBT portfolio was seemed undesirable for both the original civil servant and the manager involved. This brought to our attention the importance of an explicit role distinction in this respect.

#### Management support

As the civil servant responsible for LGBT policy had asked for advice very early on in the project, the knowledge broker had not yet worked extensively on building management support for her activities. This might also have been a reason why the collaboration stopped. Others have also shown the importance of a “deliberative approach to policy making” [36]. It was therefore decided to involve all management levels in the knowledge broker’s future activities.

### Portfolio 2: Poverty policy

September/January2011 − 2012

I. Current policy

The poverty policy was aimed at supporting people with a small income, preventing poor people from living in isolation, and strengthening the social resilience of communities. In general, the policy document acknowledged that health problems occur in combination with poverty and that disadvantaged people are therefore more likely to suffer from poor health. For more information on this association, readers of the policy document are referred to the PHS.

II Activities of the knowledge broker

As health was already on the agenda, in her meetings with the civil servant the knowledge broker endeavored to strengthen this awareness and to extend this agenda, in the hope that this would have an impact on the local poverty policy with regard to health. Knowledge brokerage included the specification—also by scientific experts—of policy alternatives that could reduce the negative effects of poverty on health and vice versa. Examples of such policy alternatives were the implementation of specific interventions for disadvantaged people and further policy integration with education sector policy.

III Impact on agenda and policies

The civil servant became more aware of and paid more attention to the relationship between poverty and health. For instance, he formulated action points on this topic, searched for information on the internet and discussed his findings with the knowledge broker (Table 1a). Subsequent drafts of the policy document included a separate section on health (Table 1b). While acknowledging that despite the presence of a PHS in Amsterdam each district is also responsible for health to some degree, this section pointed at the PHS as the organization primarily responsible in this respect. The section also referred to interventions suitable for addressing poverty and health as well as to funding made available by a health insurance company to support health interventions in disadvantaged people. Further policy integration, with either public health or other policy domains, did not occur immediately.

**Table 1.** Illustrations of findings reported in the results section

1a	The knowledge broker and the civil servant responsible for health were in close contact during the collaboration. After having read the policy document on poverty, the knowledge broker invited the civil servant to discuss this document in a meeting, together with two other experts. The civil servant agreed to such a meeting during which **the knowledge broker and the scientific experts underlined the relationship between poverty and health and specified policy alternatives that could reduce the negative effects of poverty on health and vice versa.** After this meeting the civil servant sent an email to the knowledge broker in which he formulated the central points that he had understood as being important in the relationship between health and poverty:
	“Taking health into account increases the urgency of combating poverty. Our goal is to breach the vicious circle of poverty and bad health…
	Recommendations… The municipality should make their intention to broaden the themes more concrete!”
	Expressions such as these indicate increased awareness of and consideration of the relationship between poverty and health and of policy alternatives as well as of the importance of adopting cross-sectoral policy alternatives.
	Source: email communication, 2011
1b	As a result of the activities of the knowledge broker—in terms of agenda setting and the specification of policy alternatives that could reduce the negative effects of poverty on health and vice versa—the poverty policy document was rewritten to include a separate section on health:
	“.. **Health policy**
	The previous chapter mentioned the fact that poverty is accompanied by all kinds of problems in the area of health. Research demonstrates that people in disadvantaged districts are more likely than people in other districts to have poor health and do not live as long without health problems. Within the municipality, the Public Health Service is responsible for the collective health of all people of Amsterdam**. … By combating poverty, the city district aspires to reduce deprivation when it comes to health.”** ^a^
	Source: *Uitwerkingsplan Armoedebestrijding*, Version 1.0, January 2012
	Examples of alternatives integrated into the poverty policy document:
	“A health insurance company (Agis, part of Achmea) and the municipality have established a fund to finance projects on health aimed at improving the health of households that live on a minimum income. **An example of a successful health intervention has been developed by the Public Health Service in South Limburg together with a group of dieticians entitled ‘Good food need not cost much’. People who receive debt assistance learn during meetings how they can manage to buy healthy food on a small budget. Other examples of successful interventions in the area of health promotion for households living on a minimum income can be found at** **www.Loketgezondleven.nl** **”** ^a^
1c	Some policy alternatives were not integrated into the poverty policy document because they were considered to be too scientific, too abstract, not suiting policy practice or coming from too great a distance. This is how the civil servant commented on the meeting mentioned under 1a:
	“Kirsten, What an extraordinary conversation this morning. Two worlds, each with their own language. And so far removed from each other, while we could be of great use to each other. As a result of this morning I have made a to-do list. It helps me to focus…”
	Where the civil servant speaks of two worlds, he means those of science and policy, each with their own language. This comment illustrates the policy-science gap that the civil servant experienced during his meeting with the knowledge broker and the experts who specified the policy alternatives.
1d	Examples of alternatives that were integrated into the economic policy document:
	Page 37: “The retail and hotel and catering industry … is an important element in terms of quality of life ***and in welfare”***
	Page 38 “… the growth in the number of freelance workers in the construction industry has been very strong… **This type of entrepreneur is also vulnerable**.” ^a^
	Page 42: “The scale of the informal economy is of course unknown… Because people might have the idea that certain activities should be hidden from the authorities, this holds back potential growth. This also holds back official emancipation and participation effects **and certainties such as legal protection and continuation of payment when ill**.” ^a^
	Page 42: “**The task in terms of the sectoral structure** … involves strengthening those sectors that are of importance for living conditions, livability, **welfare and health** of citizens and the investment climate…” ^a^
	Page 46: “These green and blue areas (green areas: public parks and gardens; blue areas: lakes and canals etc. K.L.) in… are… hardly known… In economic terms, these partly uncultivated and undiscovered areas in the district provide possibilities for strengthening the social climate and **the welfare and health of the inhabitants. Recreation is important in stimulating people to have an active lifestyle.**” ^a^
	Page 50 II: “The fashion industry **It is worth considering the possibility of apprenticeships.”** ^a^
1e	Example of an alternative that was not integrated into the economic policy document:
	Page 45: In the section on the hotel and catering industry, the knowledge broker asked whether it would be possible to develop policy on alcohol and happy hours. The civil servant informed her that alcohol policy is national policy and the happy hours are municipal policy (not policy at a district level).
1f	Illustration of the integration of health into the mid-term review:
	Page 17: “Not everyone is aware that taking part in sports is healthy; far from it. To reduce overweight and obesity among children, the pilot called JOGG (Young People at a Healthy Weight) was started in 2011… especially younger citizens (but also their parents!) are stimulated to start taking part in sports and to pay attention to their health. First results are positive.”
	Source: Mid-term review
1g	To support the alderman during the launch of the city-wide agreement on health between the municipality of Amsterdam and a health insurance company, an internal memo was written by the senior civil servant who was also the knowledge broker’s key informant. This document explained how the city district was allied to the city-wide agreement on health. The argument was reinforced by referring to our research.
	Summary of the content ^b^:
	The internal memo for the alderman describes the city district’s general and specific engagement with the agreement entitled “Amsterdam together for better health” [*Amsterdam samen gezonder*] which was set up between the municipality and a health insurance company. The general engagement emerges from “active integration in …policy” and through a district-oriented health policy that includes the following elements:
	- The Young People at a Healthy Weight program [JOGG] that was set up in a part of the city district;
	- **Healthy public policy research;** ^a^
	- The policy document of the PHS, which indicates that this city district was the first to develop a more district-oriented and integrated policy.
	The specific engagement of the city district with the agreement “Amsterdam together for better health” is that the city district works on more integration between care and welfare in one area of the city district.

^a^ Marked in Bold by K.L

^b^ The internal memo is an internal document and not suitable for citation of the verbatim text

IV Reflections on process factors

#### Creating trust

The creation of trust appeared to be a crucial condition for knowledge brokering. Indeed, others have previously identified both building trust and building relationships of trust as key characteristics of knowledge brokering [10, 16]. In our study we also found that it takes much time to build trust. Signing the Formal Secrecy Act appeared to be an important prerequisite in this respect. In response to questions regarding the confidentiality of information on policy provided by civil servants to the knowledge broker, the knowledge broker could refer to this formal agreement to reassure the civil servant that all information would be treated confidentially. Further trust was created through interactions that included becoming a familiar face, attending the same district meetings as the civil servant, creating a private space for exchange, and by scheduling one-to-one meetings to discuss the relationship between poverty and health, the current policy document, and possible policy alternatives.

#### Sector-specific responsibility

Sector-specific responsibilities hampered the integration of poverty policy into policies of other sectors. The importance of connecting to such responsibilities was also a key lesson in a previous study [37]. Although the civil servant agreed that the city district had a certain responsibility for health, he preferred to emphasize the responsibility of the PHS in this respect. Suggestions for further policy integration were considered not feasible because of time constraints, a mismatch with current policy practice, and incompatibility with the goals of the poverty sector, mostly formulated within the sector itself.

#### Policy-science gap

Apart from the above mentioned reasons not to integrate our suggestion in the current policy, other policy alternatives specified by the health scientists consulted were considered to be too scientific (Table 1c): they were too abstract, did not suit policy practice and came from too great a distance. However, such distance was not referred to in relation to the knowledge broker. She mostly closed the gap between science and policy by providing the civil servant with specific policy measures that matched the content of the policy document so that they could immediately be integrated. Other studies have also stated the importance of stakeholders in changing policies or programs [38, 39]. In our study, the knowledge broker further bridged the gap by scheduling well-timed follow-up meetings to discuss the actual integration of the policy alternatives and, if necessary, their adaptation. The knowledge broker was also able to bridge the gap because of her role as a boundary spanner, which meant that she could involve experts from the university and the PHS.

### Portfolio 3: Economic policy

February 2012 − September 2012

I Current policy

As from the start the economic policy document was formulated in close cooperation with local entrepreneurs, it had a broad thematic approach, in which economy was related to other sectors, such as education and environment. The policy not only addressed themes such as entrepreneurship, the labor market and education, commercial premises and the business environment, but also specific sectors, such as retail trade and the hotel, catering and service industries. Although health was not directly related to these themes, the healthcare and public welfare branches were considered because they created local employment.

II Activities of the knowledge broker

As health was not yet a topic, the knowledge broker started her meetings with the civil servant by stressing the managerial and administrative support for the activities of the knowledge broker, before going on to clarify the relationship between economy and health. Next, she endorsed the broad thematic approach of the economic policy, highlighted numerous opportunities for mentioning health implications, and suggested that these general relations be made more explicit. Specific policy alternatives included the consideration of factors such as the vulnerability of freelance workers and the uncertain position of employees in the informal economy (no legal protection or payment when ill).

III Impact on agenda and policies

The civil servant immediately understood the importance of environmental and socioeconomic determinants of health and agreed to further discussion of specific policy alternatives. Almost all alternatives specified by the knowledge broker were integrated into the policy document (Table 1d). The civil servant thought those alternatives strengthened the quality of the policy document. Rejected alternatives were those formulated at levels other than the strategic level of the economic policy document (Table 1e), for example at municipal or national level.

IV Reflections on process factors

#### Professional background of the civil servant who wrote the economic policy document

In this portfolio, creating awareness for health was eased by the civil servant’s own scientific background and experience working on the border between research and policy. Other studies have also mentioned this experience working on the border between research and policy as being one of the skills a knowledge broker should have in order to be successful [16]. In our study this facilitated her understanding of the healthy public policy concept and its application in the policy document. The civil servant was also interested in the topic of health. These factors made her willing to invest time and energy in revisions of the document.

#### Thematic policy approach

The inclusion of health as a theme in the economic policy was facilitated by the broad thematic approach of the policy, which is considered to be a key characteristic of healthy public policy [40]. This thematic approach of the economic policy document was reflected in its connections with domains such as education and environment, and allowed additional linkages with health to be made. It also allowed for further clarification of such connections in the policy document, whiles this had not been possible for the more sectorally designed poverty policy.

#### Appropriate policy level

The adoption of policy alternatives depended on the suitability of the policy level at which they were specified. Making research results suitable for the uptake in specific policies has been stressed before [38]. As the economic policy document was strategically positioned, alternatives that did not match this specific policy level were rejected. A comparison with the poverty policy portfolio also demonstrated that the level at which strategic policy documents are formulated may vary considerably within one city district, and that a knowledge broker’s policy alternatives should preferably vary accordingly.

### Policy organization

2011–2013

I Current policy

At district level, health did not fall under the responsibility of any of the policy departments. The issue of health was considered to be taken care of by the PHS. Every 4 years, the PHS draws up a municipal health policy plan. This serves as the starting point for the implementation of city-wide health policies and specific health measures per city district. Although the municipal health policy plan marked the pilot district as a priority area, so far its influence on the city district’s policies was negligible.

II Activities of the knowledge broker

Due to his former involvement with the Healthy Neighborhood Experiment, one senior civil servant was willing to become the knowledge broker’s key informant. As colleagues at the same department, they acted in close collaboration throughout the pilot. Agenda-setting for health started with two departmental meetings [Event 1; E1] to introduce the idea of healthy public policies and to clarify the presence of the knowledge broker in this respect. Like in most meetings, the knowledge broker—sometimes accompanied by a senior health scientist—explained the health-related content, while the civil servant acting as key informant clarified the pilot and its context.

At a subsequent small-group meeting [E2], a senior manager, who was head of the department, was invited to attend. Apart from agenda-setting for health, this meeting was arranged in response to the premature termination of the LGBT policy portfolio (see above), to build management support for the knowledge broker’s interactions with civil servants. The senior manager initiated a following small-group meeting [E3] aimed at persuading two city district aldermen to include health as a theme in the city district’s mid-term review. Such a review is written halfway through an administration period to justify the policy so far and to set priorities for the period to come. To achieve this aim, the knowledge broker explained the district’s health situation, while the senior manager advocated the uptake of health in the mid-term review by arguing that it should be an aspect of all city district policies.

Next, health became the subject of a departmental lunch meeting [E4]. During this meeting, which was also attended by a middle manager, the knowledge broker emphasized the importance of health for all other policy domains as well as the responsibility of each civil servant to take health considerations into account. The attendees also discussed how to adequately place the responsibility for health within the department. At the same time, the department had to produce an internal memo on all health-related city district activities [E5]. This briefing was intended to support one of the aldermen during the launch of a city-wide agreement on health between the PHS and a health insurance company. The internal memo was commissioned to the knowledge broker, but after she had made clear that this was a departmental task, the key informant made it his responsibility. In two subsequent small-group meetings [E6], the middle manager, the key informant and the knowledge broker discussed the possibilities for appointing someone responsible for health. Finally, a mini-conference [E7] was organized to emphasize health as a theme in the policies of all city districts and to announce the appointment of a temporary policy analyst for health. Two public health professors and a senior scientist of the university were invited to this mini-conference, as were civil servants, managers, two aldermen of the city district, as well as a departmental head and senior public health policy experts from the PHS.

III Impact on agenda and policies

The mere presence of the knowledge broker in the city district made some civil servants aware of their lack of knowledge on the subject of health. In the departmental meetings [E1], colleagues became acquainted with the idea of healthy public policies, which encouraged one of them to ask the knowledge broker for advice (see LGBT portfolio above). During the next meeting [E2], the senior manager acknowledged that health was an important theme for all the city district’s policies. He proposed setting up a follow-up meeting with two city district aldermen whose portfolios were the most closely related to health and then also organizing a mini-conference with representatives from the city district, the PHS and the university. When invited, the senior manager also agreed to join the advisory board of the knowledge broker pilot. During the meeting with the aldermen [E3], one alderman expressed skepticism regarding the disadvantages presented and thus the urgency of placing health on the district’s policy agenda. However, the other alderman decisively supported further agenda-setting for health while stressing the importance of also selecting specific health-related themes. With support of the senior manager, both aldermen finally agreed that health deserved attention in the mid-term review (Table 1f).

During the lunch meeting [E4], the civil servants identified connections between health and their own policy fields, such as education and participation. This made them aware of the importance of city district level policies for health, and vice versa, and of the need for more information on the topic. They told the middle manager that they were worried about the long-term lack of someone responsible for health. In response, the middle manager discussed with the senior manager the possible appointment of a civil servant for health. In the parallel internal memo for the alderman [E5], the key informant endorsed the city district’s alliance with the city-wide agreement on health, and enforced this argument by referring to the healthy policy alternatives already adopted (see policy portfolios above) (Table 1g). He also brought the need for designated staff responsible for health to the attention of the senior manager. In response, the senior manager proposed the temporary appointment of a policy analyst for health. Further discussions of this idea [E6] led to the candidacy of the senior civil servant who acted as key informant.

At the mini-conference [E7], the public health professors explained what healthy public policies are, while the civil servants stated the links they had previously identified between health and their own policy fields. Although some doubts were expressed with regard to how the integration of health in other policies would be specified and financed, the idea of healthy public policies was generally approved. The appointment of a temporary policy analyst for health was widely appraised as it met a previously felt need. The policy analyst’s first assignment was to optimize healthy public policies within the district’s administrative and political context, by choosing relevant health themes, formulating health objectives and specifying health ambitions.

IV Reflections on process factors

#### Academic collaborative center context

The fact that the knowledge broker pilot was set up in the context of the ACC helped to make it a success. The boundary-spanning properties of this public health partnership created long-lasting commitment between the stakeholders, namely the city district, the PHS and the universities. Similar findings from another study have demonstrated the importance of a boundary organization that connects science and policy [22]. In our study, this commitment is illustrated by the inclusion of the senior manager on the knowledge broker pilot’s advisory board and the active role of the professors at the mini-conference. The long-lasting commitment provided the knowledge broker with a double role, which allowed her to span the boundary between the city district and the universities, and to establish a long-term presence in the city district, thereby creating opportunities to raise the matter of health at every possible occasion. This need of long-term presence of the knowledge broker in the field was also a key lesson in the intervention described by Dobbins et al. [16].

#### Professional background knowledge broker

The fact that the knowledge broker had a professional background in policy making was a prerequisite for successful agenda-setting. For instance, this enabled her to understand the standard procedures in policy development and the accepted routes for administrative decision making. According to Sauerborn et al. [39], researchers’ lack of knowledge of the policy-making process is one of the reasons why research is frequently not used. In our study, the opposite is illustrated by the knowledge broker’s use of the mid-term review to set the agenda for health at the administrative level. A second benefit of her professional background was her understanding of the authority of each of the civil servants, managers and aldermen. This is illustrated by the deliberate casting—in accordance with each person’s qualification—of who would introduce the pilot, who would explain the health-related content, and who would advocate health in the various meetings. A third advantage was her ability to anticipate stakeholders’ responses and to carefully plan the knowledge brokering activities accordingly. This is illustrated by concurrently involving both civil servants and a middle manager in the discussion of where to place the department’s responsibility for health, which resulted in sufficient bottom-up pressure for the senior manager to appoint a policy analyst for health.

#### Various policy levels

Inspired by her awareness of how different policy levels interact, the knowledge broker was active at various levels at the same time. The necessity of such interaction at different policy levels at the same time has been demonstrated previously [37]. In our study this resulted in the city district stakeholders gradually taking over the initiative to create healthy public policies. In this way we can speak of a ripple effect, in that a specific development that is initiated from the outside, becomes an independent phenomenon that is able to start from within [41]. This is illustrated firstly by the fact that the civil servant who was the key informant*—*on his own initiative*—*directly discussed the district’s responsibility for health with the senior manager, and secondly by the fact that the senior manager initiated meetings in this respect and proposed the appointment of a policy analyst for health. Other examples of such reversed initiatives are requests for advice on health aspects of city district policies that were not central to this knowledge broker pilot, for instance mobility and socioeconomic projects.

## Discussion

### Summary of the main findings

We employed a knowledge broker in an Amsterdam city district to create healthy public policies by setting the agenda for health and by specifying evidence-informed policy alternatives [18, 19]. The knowledge brokering strategy we applied was especially successful in agenda-setting for health, but also led to the adoption of policy alternatives in individual policy portfolios. In other words, health became an issue on the formal policy agenda, as illustrated by its uptake in the city district’s mid-term review and the appointment of a policy analyst for health. Our study corroborated the importance of process factors such as building rapport and trust [11, 17], clearly defining the knowledge broker role [17], and providing sufficient management support [37]. We also saw the benefits of multilevel agenda-setting [19] and specifying easy-to-adopt policy alternatives at appropriate policy levels [40]. Although sector-specific responsibilities sometimes hampered the adoption of cross-sectoral policy alternatives [37], we also found that thematically designed policy documents were particularly useful in providing opportunities to create healthy public policies [40]. In terms of strategy for developing healthy public policies, our results demonstrate that the knowledge broker is a potential key asset in developing essential elements for healthy public policies. By corroborating these elements in another policy context, our case study may have a general value and therefore be relevant to other pilots involving a knowledge broker. Further interpretation of our results revealed three additional themes in knowledge brokering: the importance of boundary spanning [42], the prospects of a ripple effect [41], and the added value of participant observation.

### Boundary spanning

Successful knowledge brokering depends on spanning the boundary between research and policy effectively [9, 16]. In our pilot study, a precondition in this respect was the dual position of the knowledge broker and her work experience in both research and policy. Because of her double background—in both policy and research—she knew all the “informal and formal norms of the relevant organizations, as well as their internal operations and politics” [42:84]. Being present in, acquainted with, and accepted by both these worlds minimized the insider-outsider tension that often prevents successful knowledge brokering across boundaries [35]. Our results show how the minimization of this effect may support all three dimensions of knowledge brokering [12, 15] Being an objective outsider helped the knowledge broker to create conditions for effective knowledge management, such as trust and management support [9, 12]. Acting as a boundary spanner enabled her to involve outsiders from the university and the PHS, thereby strengthening linkage and exchange. Being an involved insider gave her access to decision makers, which then helped her to move the issue of health onto the formal district agenda, and thus build further capacity [12, 18]. We believe that the ACC context, with its long-lasting commitment, was a crucial condition in this respect: it provided a particular setting that benefitted the brokering of knowledge across boundaries [11:119].

### Ripple effect

A second explanation for successful agenda-setting was the occurrence of a ripple effect within the city district [38]. A ripple effect means that a certain movement that is initially initiated from the outside, such as agenda-setting for health, becomes an independent phenomenon that goes on to grow from within [41]. In knowledge brokering such a ripple effect may be comparable with reaching “critical mass” [16], which is believed to be only possible if the strategy involves multiple participants throughout an organization [16]. From a policy perspective, such a ripple effect may mirror “spillover”, or the chain of events that occurs when one “window” for setting an agenda opens another one, and so on [19]. In our study, the ripple effect is best illustrated by the reversal of the initiative to create healthy public policies − this gradually shifted form the knowledge broker to the city district stakeholders. Apart from the supportive role of boundary spanning in this respect (see above), we believe that the ripple effect was especially brought about by our multilevel approach, in which we deliberately involved different policy levels [16].

### Participant observation

Successful knowledge brokering requires a customized approach that takes context into account [8, 9, 12, 16]. Our study illustrates how a developmental evaluation with an action research approach [27, 28] can benefit from the application of the method of participant observation [20]. We applied this method by combining the roles of knowledge broker and action researcher in the hands of one person. This created opportunities for directly tailoring our knowledge brokering strategy to experiences and contextual factors in the field, as well as for identifying different types of outcomes of the complex social activity that knowledge brokering is [12]. In doing so, we went through a “cyclical process of fact finding, action, and reflection, leading to further inquiry and action for change” [35:686]. We illustrate this cycle in the results section by reporting the activities and impacts of the knowledge broker and comparing them with relevant reports from the literature to critically reflect on the essential process factors. Judith Okely refers to this cycle of action as follows: “The fieldworker cannot separate the act of gathering material from that of its continuing interpretations” [31: 20-21]. In other words, the “fieldworker” thinks and rethinks over his or her material and when analyzing the data compares it to other scientific writings on the subject [31]. In our study, this cycle of action and learning helped us to identify boundary spanning and the ripple effect as two underlying principles of transferring knowledge into action [12].

### Limitations

This study has several limitations. A first limitation is that we studied only a single knowledge broker case. Findings of such single-case studies are usually regarded as context-specific and hard to generalize. However, the participant observation method partly compensates for this limitation [20]. The decontextualizing empirical and theoretical reflections on critical process factors resulted in an understanding of the knowledge broker approach that may be useful for future initiatives and evaluations. A second limitation, restricted objectivity, is introduced by the same participation observation method, in which the observer is also the primary tool of research [20]. We believe however that our use of multiple data sources, our detailed description, including illustrative excerpts from the materials collected, and our close collaboration with stakeholders within the city district, have all helped to prevent misinterpretations by the knowledge broker-researcher. In addition to this, as we were aware of the potential bias of field notes—given the dual role of the researcher—we also asked the key informant of the city district to read and approve this manuscript. A third limitation was that our study involved only a single knowledge broker. Despite her dual position and her work experience in both research and policy, some insider-outsider tension still remained: the knowledge broker was specifically not expected to interfere in activities that were typical of the policy domain. However, she managed to balance the need to be involved and yet detached by incorporating frequent briefings and debriefings with the city district’s key informant into the cyclical action and learning process [35]. A final limitation is related to the question of what “success” in knowledge brokering actually is [12]. In the process of policy development, we saw that the issue of health gained formal agenda status and was supported by positive decisions made by the authorities. Although we know that effective policy implementation will not automatically follow [18], these findings allow us to conclude that our knowledge broker approach noticeably increased the numbers of opportunities for strengthening the city district’s policies on the determinants of health and thus for creating healthy public policies.

## Conclusions

The employment of a knowledge broker who works simultaneously on agenda-setting for health and the specification of health-promoting policy alternatives seems a promising first step in establishing local healthy public policies. Future studies are needed to explore the usefulness of our approach in further policy development and policy implementation.

### Availability of supporting data

The data from this study is stored at the Department of Public Health at the Academic Medical Center, University of Amsterdam, the Netherlands. The data is available for researchers seeking collaboration (with the permission of the research committee).
